# Difficulties in using Oswestry Disability Index in Indian patients and validity and reliability of translator-assisted Oswestry Disability Index

**DOI:** 10.1186/s13018-015-0230-8

**Published:** 2015-06-09

**Authors:** Janardhana P Aithala

**Affiliations:** Kasturba Medical College, Mangalore (Manipal University), Mangalore, India; Department of Orthopedics, KMC Hospital, Attavar, Mangalore, 575001 India

**Keywords:** ODI, Low backache, Disability, Outcome assessment

## Abstract

**Background and aim of the study:**

In Indian patients, in view of language plurality and illiteracy, self-reporting of English version of Oswestry Disability Index (ODI) is not practical. Our study aim was to find out to what extent self-reporting of ODI was possible and in cases where self-reporting was not possible, to see validity and reliability of a translator-assisted ODI score.

**Materials and methods:**

Fifty patients with low backache and who could not use the English version were assessed with ODI with the use of two translators at a gap of 3 h in a test and retest manner. Patients were also asked to report the most important disabling activity in their day-to-day life.

**Results:**

A total of 58 questionnaires were filled during the study period out of which eight patients (14 %) self-reported English version; while 50 patients needed a translator. The Cronbach’s alpha between two translators for the ODI scores of 50 patients was 0.866, but aggregate of difference between two scores for each ODI component shows high difference between two translators for question nos. 3, 9, and 10. Cronbach’s alpha was best when item no. 3 was deleted (0.875, translator 1; 0.777, translator 2). Thirty-seven people did not answer the question related to sexual activity. Agreement between two values was assessed using Kendall’s tau and was found good (0.585, Spearman’s coefficient 0.741). Kendall’s tau values correlating total ODI score and individual components show that all the items move together, but correlation was poor for question no. 3 (*P* value 0.16 for translator 2).

**Conclusions:**

Translator-assisted ODI is a good outcome assessment tool in backache assessment in places where validated local language versions are not available, but in Indian patients, inclusion of question nos. 3 and 8 related to weight lifting and sexual function needs to be reviewed.

## Background

Oswestry Disability Index (ODI) [1] is a commonly used condition-specific outcome measure for outcome assessment in low backache. This tool was developed by J Fairbank and others in 1980 and later had been tested by various authors for its reliability and usefulness [1–3]. ODI has been considered as a good instrument for low backache-specific disability assessment as it addresses both pain and function [4]. ODI has been translated into many languages and have been validated in each of these languages [5–11]. Recently, ODI has been translated and validated in one of the Indian languages, too (Marathi version) [12].

ODI is a self-reported questionnaire containing 10 items. Each item is scored from 0 to 5. Both paper and electronic versions have been used [13]. However, in Indian patients, we have found that there are certain difficulties in using this questionnaire. Many of our patients do not understand English, and we do not have any validated local language translations, although recently, Marathi version has been validated [12]. In India, there are more than 30 languages, and translating them into a particular language may not benefit all, there are also patients who cannot read any of the languages. Use of translator in ODI has not been described in literature, nor validated. In addition to the difficulties associated with self-reporting, the questionnaire items are usually designed for western population and our activity patterns are entirely different. Our patients need to squat, sit cross legged, work in floor, and work in fields with uneven surface. So there are concerns whether this questionnaire accurately predicts our disability patterns. Since ODI has been tested and validated, it may not be wise to leave out this disability assessment tool completely; instead, we believe we should focus on modifying this index for our needs. Hence, we decided to study the problems related to scoring and then find out what items are relevant in our set up and what items need modifications. Such an exercise will need multiple studies and participation by multiple centers, but we thought we will make a beginning that will provide an insight into problems related to ODI scoring in our population. We do not find any literature regarding difficulties in the use of Oswestry Disability Index in Indian patients before. In this part of study, we would like to address issues related to use of ODI due to language constraints and see how use of an instructor/translator can help in such circumstances. We would also like to see how individual items correlate with patient’s overall disability and to what extent the scoring system represents most important disability of our patients.

## Materials and methods

### Objectives of the study

This part of our study aims to know the number of patients who can self-report the questionnaire, to know the validity and reliability of translator-assisted ODI scores (as this could be only way to get the scoring) by looking at the agreement between two values, and also to see how individual items show internal consistency and relationship with overall disability of patient when a translator was used. The study also aims to see how much the existing ODI components are valid in Indian patients.

### Patient selection

This study was authorized by local ethical committee (Manipal University Ethics Committee, UEC/06/2013–2014 dated 25 March 2014). Study included all patients presenting with low backache irrespective of diagnosis and treatment modalities used. Patients should be willing to wait for 3 h to take a retest after the initial evaluation (test). Patients with trauma, patients who were suspected to have infections and tumors, and patients with acute severe pain with inability to sit and walk were excluded from the study. Similarly, patients who were not willing to fill the questionnaire were excluded from the study. Initially, patients were asked to fill the questionnaire (English version 2.1a) independently, when they are unable to fill the questionnaire in view of inability to read the English version, a translator was provided whose role was limited to translating the individual items and leaves the choice of answer to patient. A resident who has been trained in using Oswestry Disability Index and who knows the local language was the first translator. Initial 10 cases done by the resident were excluded from the study. After 3 h, the questionnaire was filled with another translator. The second translator was the author who himself is a spine surgeon, knows the local language very well, and also was using Oswestry Disability Index for many years.

### Scoring method

Each section was scored from 0 to 5. Index was obtained by adding all the scores and then dividing by the highest possible score from all the items the patient answered and then multiplying it by 100. If the patient answers all the questions, the denominator is 50, while if the patient has not answered one question, denominator becomes 45 and so on.

After filling the questionnaire, the patient was asked to fill up what the most important and most disabling activity/activities in their day to day life is/are. In addition, the author had an opportunity to discuss the difficulties he faced while answering the questionnaire. This part was done only after he filled the questionnaire completely, Oswestry Disability Index questions were asked again, and the patient was asked if he had any difficulty in answering each of these questions. This step was included just to understand whether these activities described in the questionnaire are relevant in their day-to-day activities. Findings or observations of these may not be helpful in performing a statistical analysis. This step was kept last after the questionnaires were filled with the help of both the interpreters as there will not be any bias while filling the questionnaire, and our observations were discussed in the Discussion section.

Our statistical analysis included incidence of patients who could do self-reporting, incidence of patients who could use the local language if available, measurement of internal consistency of values using Cronbach’s alpha, Spearman’s correlation, and Kendall’s tau values between individual components as well as total ODI scores between two translators to highlight the agreement between two translators, correlation between total score, and individual components of ODI using Kendall’s tau and Cronbach’s alpha (including values when a particular item was deleted). SPSS version 16 was used for statistical analysis.

The author does not have any competing interest and did not receive any external funding for this study. The study was performed according to Helsinki declaration, and ethical committee clearance was taken from Manipal University Ethical Committee.

## Results

A total of 58 questionnaires were filled during the study period. Eight patients could fill the questionnaire themselves. Thus, 14 % of the patients could do self-reporting. The remaining 50 patients were helped with a translator. Our target of 50 patients was based on 80 % power with reference value of *r* = 0.52 (Fisher K and Johnson M et al. [3].) with 95 % confidence limits. These patients underwent one more session of scoring with the help of another translator after 3 h.

Among the 50 patients, 19 patients could not read even the local language. The geographical area from where we have included patients for the study were from western coastal area of Karnataka state (Udupi district) and was considered to be having a high literacy rate compared to the other parts of the country. The following table (Table 1) shows salient features of baseline data.

**Table 1:** Baseline data

Baseline data	Values
Mean age	51.57
Male/female ratio	15/35
Percentage of people who are illiterate	38 % (19/50)
Number of questions unanswered	40
	Question no. 8: 37 people
	Question no. 3: 1 person
	Question no. 7: 1 person
	Question no. 10: 1 person
Clinical diagnosis	Mechanical backache: 2
	Intervertebral disc prolapse: 11
	Lumbar canal stenosis: 14
	Spondylolisthesis: 13
	Postoperative status: 9
Mean ODI Score: (translator 1)	35.22
	Categorisation [22]
	Mild (0–20) - 10 patients
	Moderate (21–40) - 23 patients
	Severe( 41–60) - 13 patients
	Crippled (61–80) - 4 patients
	Bed ridden (81–100) - 0
Mean ODI score: (translator 2)	34.80
	Mild - 9 patients
	Moderate - 23 patients
	Severe - 17 patients
	Crippled - 1 patient
	Bed ridden - 0

Question no. 8 was not answered by 37 patients. Question nos. 3, 7, and 10 were not answered by one patient each.

**Fig. 1:**
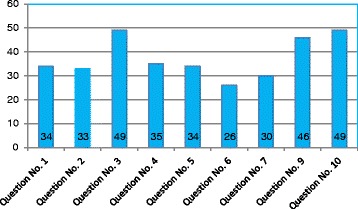
Shows the aggregate of difference between two translators in each of ODI components

**Fig. 2:**
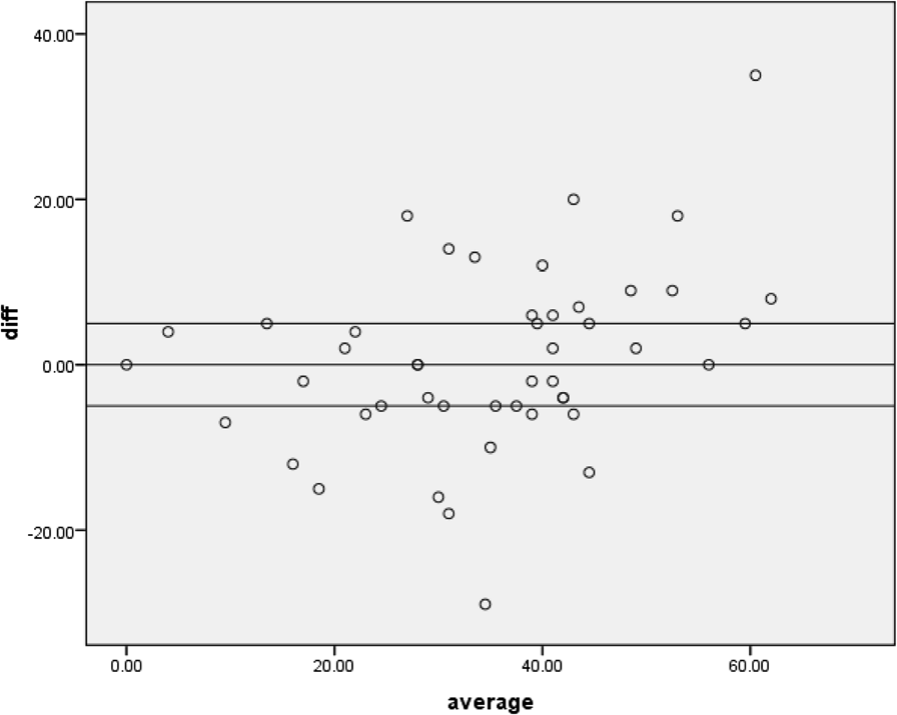
Shows the difference between two translators and average between two translators. (*lines* above and below 0 mark are set at 5 points and *dots* above +5 and −5 line indicate difference of more than 5 points between two translators)

Internal consistency was measured with Cronbach’s alpha; the Cronbach’s alpha values of ODI between two translators were 0.866 indicating good internal consistency. Agreement between two translators was measured using Spearman’s correlation and Kendall’s tau. Table 2 shows correlation coefficient values of ODI and individual components of ODI scores between two translators. It was only in question no. 2 that there was poor agreement (less than 0.5), in both Spearman’s and Kendall’s tau correlation between two translators. Question no. 3 showed poor agreement with Kendall’s tau correlation between two values. We also analyzed the agreement between two values by looking at aggregate of differences between two values, and Fig. 1 shows aggregate of differences between two values while, Alderman Scatter gram (Fig. 2) shows difference and average between two translators. Question nos. 3, 9, and 10 show higher difference between two translators.

**Table 2:** Correlation of ODI scores and individual item scores between two translators (scores above 0.5 are considered good agreement)

Scores	Kendall’s tau	Spearman’s coefficient
*Total ODI*	*0.586*	*0.741*
Question no. 1	0.46	0.52
Question no. 2	0.275	0.297
Question no. 3	0.43	0.51
Question no.4	0.5	0.58
Question no. 5	0.59	0.65
Question no. 6	0.73	0.81
Question no. 7	0.49	0.54
Question no. 9	0.48	0.56
Question no. 10	0.50	0.60

Italicized data is for total ODI score while the rest are individual ODI components

### Relevance of ODI questions in our patients

Cronbach’s alpha was calculated for both ODI values separately by deleting each item, and Table 3 shows the values. Although there is no significant difference in values with deletion of one of the items, values are highest when item no. 3 was deleted and this was noticed for ODI values obtained with both translators. Next, we correlated total ODI score with individual components. Kendall’s tau values correlating total ODI score and individual components are shown in Table 4. Almost all the items move together (*P* value less than 0.05 indicating significantly good correlation) except question no. 3 (Translator 2, *P* value 0.16).

**Table 3:** Cronbach’s alpha values when a particular item was deleted (excluding question no. 8)

ODI individual questions (deleted)	Translator 1	Translator 2
One	0.862	0.72
Two	0.864	0.728
Three	0.875	0.777
Four	0.855	0.707
Five	0.869	0.734
Six	0.857	0.685
Seven	0.871	0.736
Nine	0.858	0.704
Ten	0.851	0.71

**Table 4:** Correlation between ODI and individual questions using Kendall’s tau

ODI questionnaire items	Translator 1		Translator 2	
	Kendall’s tau	*P* value	Kendall’s tau	*P* value
One	0.531	<0.001	0.467	<0.001
Two	0.622	<0.001	0.396	<0.001
Three	0.458	<0.001	0.259	0.016
Four	0.649	<0.001	0.477	<0.001
Five	0.468	<0.001	0.398	<0.001
Six	0.6	<0.001	0.512	<0.001
Seven	0.547	<0.001	0.418	<0.001
Nine	0.643	<0.001	0.55	<0.001
Ten	0.661	<0.001	0.519	<0.001

After completing the questionnaire, the author sat with the patient and discussed the difficulties he had during filling of the questionnaire, and also, he was asked to mention the most difficult activity/activities in his day-to-day life. We made some important observations. Walking was the most difficult thing in 22 patients, bending forwards in 13, getting up from floor in 16, sitting cross legged or squatting in 12 patients, walking on uneven surface in 2 patients, night pain in 2 patients with difficulty in sleeping, and giving away of knee due to weakness in 2 patients(Table 5). While walking and pain-disturbing sleep were represented in ODI, remaining activities which were noticed in a total of 26 patients were not represented in the disability questionnaire. This clearly shows that the disability index does not represent most disabling activity of a person.

**Table 5:** Shows most difficult activity in day-to-day life and their representation in ODI questionnaire

Most difficult activity in the day-to-day life	Number of patients	Representation in Oswestry Disability Index
Walking	22	Question no. 4
Getting up from floor	16	Not represented
Bending forwards	13	Not represented
Sitting cross legged and squatting	12	Question no. 5 but not specific
Walking on uneven surface	2	Question no.4 but not specific
Night pain with difficulty in sleeping	2	Question no. 7
Giving away of knee due to weakness	2	Not represented

## Discussion

Our first aim of study was to find out whether self-reporting is possible in India. In our study, only 14 % of patients could self-report using the English version, while 38 % were unable to self-report even if the questionnaire was available in the local language in view of their inability to read any of the languages. We did a Medline search and found seven articles from Indian authors with two of them in Indian journal of orthopedics. One of the articles [14] quotes 97 % of the reporting of the questionnaire but does not specify what version was used. However, our results show that it will be impossible to achieve such high questionnaire filling unless there is local language translation or help of an interpreter in our area. Validated versions in Indian languages are not available in most of languages except Marathi [12] which too was available only recently. We looked at the Indian literacy rate and ability to read the English language to see how it varies or how it is similar across different parts of India. The Indian literacy rate is 74.04 % [15] which varies from urban and rural areas. Udupi, the place where we conducted this study, is a semi urban area and has a literacy rate of 86.24 % which is better than national average, but still there are 14 % people who may not be able to read the questionnaire in any of the languages. With regard to the use of the English version, India has 20.68 % [16, 17] English speakers while the rest cannot speak English; hence, the English version cannot be self-reported in close to 80 % of the patients. Majority of patients with degenerative disorders belong to higher age groups, and educational levels in these are still poorer than national averages. This is the reason why in our study, the number of people who used the English version and the number of people who would have been benefited from a translated version in the local language are still less than national averages. However, on the brighter side, majority of schools in India now teach in English, and hence, in the future, there is a possibility that English versions of the questionnaire can be used in majority of patients.

In addition to the problem of illiteracy coming in the way of using a questionnaire translated into the local language, we have one more concern regarding translation of a questionnaire into the local language. In India, we find people speaking different languages; even in a small place like Udupi, we have people speaking two different languages. So local translations should be done in all these languages and needs to be validated as specified by Fairbank et al. [1]. This is a herculean task.

Use of translator is not described earlier; we decided to look into these aspects. PubMed search did not yield a single paper quoting use of translator, but Google search showed one of the thesis papers done in South Africa where similar conditions like language plurality is present. According to Christelle Grebe [18], “Making use of a translator may not be the most reliable form of data collection but keeping in mind the literacy levels in South Africa, it may be a more accurate way of collecting correct data from individuals compared with incurring massive costs in translating the documents into a target language.” We also found that a patient will have confusions regarding the questionnaire, and when a translator who has clear understanding of Oswestry Disability Index explains clearly, patient can understand more clearly than they could understand by reading themselves. For example, for item no. 3 (weight lifting), if a patient can lift weights placed on a table, it should be scored as two or three, while ability to lift weights from floor should be scored either 0 or 1. For a labor who is used to carry heavy loads, which he always does by keeping it over an elevated surface before lifting, he may score either 0 or 2 depending on how he understood the question. Similarly, if he can lift moderate weights, he will mark 3 even if he could do so from the floor as the response carrying 3 points is the only response which talks about light to medium weights. The confusion is that the item does not specifically explain the quantity of weight in these sections and that explanation was necessary sometimes to clear the doubt; this was possible only when a translator was available. Better Cronbach’s alpha values (with item deleted, Table 3) seen with interpreter 1 (compared to translator 2) who is the senior person and belongs to local area also supports this. We also noticed certain problems specifically with item number 3 as many people scored this item, but when we sat with the patient and discussed with the patient, they explained that their scoring was arbitrary as in their day-to-day life, there is no need to do any weight lifting. This indicates that they could have answered with speculation rather than actual performance. One of the solutions could be not to score this item at all and calculate the ODI [19] with only nine or even eight items, as many people did not answer the question related to sex function. We also found that Cronbach’s alpha value is highest both with translator 1 and translator 2 when item no. 3 was deleted although it is only a marginal improvement.

Majority of patients did not answer question no. 8 which is related to sexual function. We believe that this could be due to cultural and ethnic background of Indian population, and people may not answer although there could be some disability related to the same. Other explanation may be, as most of patients with degenerative disc disease are older people, this question might have been irrelevant in Indian context. Probably this is the one question where presence of a translator had a negative effect in getting the answer.

Our second question was whether use of a translator leads to bias. We selected two translators in such a way that one of the translators is not involved in treatment process. One of the residents was trained in Oswestry Disability Index and was advised to perform the roll of translator. We found that there is a good agreement as seen from Cronbach’s alpha; thus, our statistical results show that use of translator can be a good option rather than converting ODI into all the languages in a country like India with so many languages as well as people with illiteracy. We conducted our study in a way similar to test retest hypothesize conducted by Fairbank et al. (1980) in which the questionnaire was completed on two consecutive days, and Baker, Pynsent, and Fairbank et al. 1989 [13], in which paper and computerized versions were used on the same day. In our study, it was a similar situation in which we tried to see whether there will be consistency between different people interpreting and explaining to patient the questionnaire items. The correlation was excellent as seen in Table 2, using Spearman’s coefficient and Kendall’s tau, indicating that people can interpret or translate and explain to a patient clearly and get the scoring, which will be very useful when a particular language version is not available or patient is unable to read and write any language. One of the prerequisite is the person who translates should be good with the local language, and it may be ideal to train a person who is not directly involved in the treatment. This could probably lessen the bias arising from surgeon’s preference to see some form of treatment getting favoritism. In an institutional set up in India, it is very much possible to train a person and involve in helping the patients to score ODI.

While the correlation between total ODI scores of two translators was good, we looked at individual items; we found that some of the items had significant variation between two translators. While, Kendall’s tau and Spearman’s correlation showed that there is a poor agreement for question no. 2 (0.27 and 0.29, respectively) and question no. 3 (Kendall’s tau 0.43), aggregate of difference between two values as shown in Fig. 1 indicates that item nos. 3, 9, and 10 showed high variations between two translators. This was also seen from Cronbach’s alpha when calculated separately with particular item of interest deleted. Although most of the items of Oswestry Disability Index correlates well with total ODI score, question no. 3 related to weight lifting do not correlate well with total ODI score (for translator 2), as Kendall’s tau values are slightly lower (0.259, *P* 0.016). In all these tests, question no. 3 does not correlate well compared to other items. As already mentioned, there is a strong possibility that some of the patients speculated while scoring these items. This observation was based on the discussion author had with the patient after the completion of scoring. These observations were backed by Table 5 which shows most important disabling activities in their day-to-day life. None of the patients were bothered with their ability to lift weights, traveling, or social life. Although it is difficult to prove statistically which one of the translators is right in eliciting a correct response, a well-trained person can explain clearly the scoring system to patient and get a more or less correct response; it is also possible that the questionnaire pattern and their non-representation in their day-to-day life may lead to significant bias. We have also noticed that some patients could not accurately tell how much traveling they can do as they need not travel every day. Once again, we refer to our observations in Table 5. Only in 24 patients (walking in 22 patients, difficulty in sleeping due to pain in night in 2 patients) that at least one of the most important disabilities they face in their day-to-day life is represented in the questionnaire, but in the remaining patients, their most important disability is not represented in the questionnaire. Fisher and Johnson [3] also found that lifting weights and actual performance did not correlate and found that weight lifting do not match actual performance. Our impression is these are the items which may not suit our people’s activities and hence subjected to higher bias between two translators, while use of translator should not be a problem as the statistical values were good for most of the questions and poor correlation was specific to those questions which represent activities that are not necessarily required in their day-to-day activities.

It would be tempting to replace few items like question nos. 3, 8, and 10 with some other questions based on most important activity that they would like to get corrected by treatment. There was one study from ICMR [20] in which question related to sexual function was replaced by house making, although this study was an epidemiological study and was aimed at finding out the incidence and prevalence of spinal disorder in general. ODI has been an excellent tool for comparing different conditions and also comparing different treatment interventions [21]. Hence, it would be easy to use the same ODI score with minor modifications to suit our people’s demands rather than trying for an entirely different scoring system. The modifications which we consider need careful evaluation and validation through further multicenter tails and we would like to address these issues in our subsequent research.

Thus, to summarize, we believe translator use is a good option in countries where validated versions in local language are not available. But certain questions may have to be modified in countries like India, where activities of people are different from the west. While this paper gives only an insight into these problems, more specific work needs to be done preferably at multiple centers to find out which activities are relevant for our patients and then try to incorporate them into the questionnaire and test their validity. We would also like to test the validity of the questionnaire with an interpreter used in predicting treatment response. We already have started some work on these issues and will be coming out with our results soon.

## Conclusions

In the Indian context, it would be difficult to use the English version of ODI or even translated version in all patients and it needs the help of a translator. The use of a translator in such situations and use of ODI is possible and shows good consistency and reliability. However, in the Indian patients, inclusion of certain questions like weight lifting and sexual life needs to be reassessed as these activities are not required in day-to-day life and are likely to be incorrectly answered or omitted by certain patients.
